# Methodological Considerations in PRP Treatment for Lichen Planopilaris: Addressing Potential Bias

**DOI:** 10.1111/jocd.70157

**Published:** 2025-04-01

**Authors:** Mathias Willaert, DirkJan Hijnen, Rick Waalboer‐Spuij

**Affiliations:** ^1^ Department of Dermatology Erasmus MC University Medical Center Rotterdam the Netherlands

**Keywords:** cicatricial alopecia, clobetasol, hair loss, inflammatory hair disorder, lichen planopilaris, platelet‐rich plasma

Dear Editor,

We are writing in response to the recent publication titled “Platelet‐Rich Plasma as a New and Successful Treatment for Lichen Planopilaris: A Controlled Blinded Randomized Clinical Trial” by Behrangi et al. (2024) in the *Journal of Cosmetic Dermatology*. The study contributes valuable data on the use of platelet‐rich plasma (PRP) for lichen planopilaris (LPP); however, we wish to address a significant methodological issue related to performance bias that could have influenced the study's outcomes.

The study design involved a single‐blinded approach, where only the outcome assessor was blinded to the treatment groups. Nonetheless, the participants were aware of whether they were receiving PRP in addition to clobetasol or clobetasol alone. This lack of blinding among participants introduces a risk of performance bias. This type of information bias is particularly significant in a condition like LPP where subjective symptomatology—such as pruritus, pain, and burning—plays a crucial role in outcome assessment.

Patients aware of receiving what is perceived as a more novel or advanced treatment (in this case, PRP) might expect greater improvement and report better outcomes. This expectation can influence their subjective reporting of symptoms, potentially leading to an overestimation of the treatment's effectiveness. Conversely, patients in the control group might report less improvement because of lower expectations, even if they experience similar physiological benefits. The psychological impact of knowing one's treatment group can significantly affect patient‐reported outcomes, which are central to studies involving chronic conditions with a subjective symptom burden.

Moreover, the Lichen Planopilaris Activity Index (LPPAI) used in this study, which heavily relies on patient‐reported symptoms, is particularly vulnerable to such bias. The differences in symptom reporting between the PRP and control groups could skew the perceived efficacy of PRP, thereby complicating the interpretation of the study's findings. The study's conclusion that PRP is superior to clobetasol in managing LPP might be partially attributable to this bias rather than the treatment's inherent efficacy.

To mitigate performance bias, a double‐blinded design where both participants and assessors are unaware of the treatment allocation would address this issue. This approach would reduce the influence of patient expectations on symptom reporting, providing a clearer picture of the true efficacy of PRP in treating LPP.

In light of these concerns, we recommend that future studies on PRP for LPP or similar conditions employ a double‐blinded methodology to ensure more reliable and unbiased results. Such a design would enhance the validity of the findings and support more robust conclusions about the efficacy of PRP in clinical practice.

## Author Contributions

**Mathias Willaert:** conceptualization, methodology, writing – original draft preparation. **DirkJan Hijnen** and **Rick Waalboer‐Spuij:** conceptualization, supervision.

## Ethics Statement

The authors have nothing to report.

## Conflicts of Interest

D.J.H. served as the investigator for AbbVie, Almirall, Galderma, LEO pharma, and Sanofi, and as a consultant for AbbVie, Eli Lilly, Galderma, Pfizer, and LEO pharma. M.W. and R.W.‐S. declare no conflicts of interest.

## Data Availability

The authors have nothing to report.

